# The Growing Concern of Unfilled Interventional Cardiology Fellowships: Causes, Trends, and the Path Forward

**DOI:** 10.1016/j.jscai.2026.105311

**Published:** 2026-04-05

**Authors:** Khalid Sawalha, Munes Albadaineh, Muhammad Ali Zulqarnain

**Affiliations:** a Division of Cardiovascular Medicine, University of Arkansas for Medical Sciences, Little Rock, Arkansas; b Division of Cardiovascular Medicine, Mercy Health, Toledo, Ohio

**Keywords:** clinical cardiac electrophysiology, electrophysiology, fellowship, interventional cardiology, lifestyle, match, unfilled positions

## Scope of the problem

In 2025, interventional cardiology (IC) fellowships achieved a historic milestone by participating in the National Resident Matching Program (NRMP) following a successful collaborative effort among Society for Cardiovascular Angiography & Interventions (SCAI) leadership, the Match Task Force, training program directors, and faculty.^1^ However, the 2025 IC Match results released by the NRMP have raised concerns across the IC communities. For the second consecutive year, a notable number of fellowship positions remained unfilled nationwide, prompting program directors, training institutions, and professional societies to reassess the future of this field.^1^ For example, in 2024, a total of 164 programs participated in the match, offering 326 positions, with a match rate of 94%. The following year, 156 programs offered similar positions, with a match rate of 83.4%.^1^^,^^2^ No longitudinal match data are yet available to assess year-over-year trends due to the recent NRMP participation. Before 2024, most IC programs filled fellowship positions outside the NRMP process, making it difficult to accurately compare historical fill rates or evaluate long-term trends in applicant interest.^1^^,^^2^ Although IC has long been regarded as one of the most appealing and technically demanding cardiology subspecialties, the recent decline in fellowship applications suggests that its allure might be fading.^2^ In sharp contrast, other subspecialties such as electrophysiology (EP) have seen growing interest among applicants, reflecting a paradigm shift in physician priorities—from procedural intensity to lifestyle balance, career sustainability, and financial return on investment as seen in Table 1.^2^, ^3^, ^4^, ^5^

**Table 1:** Showing annual program participation, position availability, and match rates for US electrophysiology and interventional cardiology fellowship programs

Year	Programs participating		Positions offered		Match rate (%)	
	Electrophysiology fellowship	Interventional Cardiology fellowship	Electrophysiology fellowship	Interventional Cardiology fellowship	Electrophysiology fellowship	Interventional Cardiology fellowship
2019	83	–	130	–	62	–
2020	78	–	135	–	85	–
2021	82	–	129	–	96	–
2022	77	–	130	–	95	–
2023	84	–	132	–	98	–
2024	89	164	145	326	96	94
2025	98	156	152	326	98	83

Data are presented by match year and include the number of participating programs, total positions offered, and overall match rate (%). Interventional cardiology data are unavailable prior to 2024. These data were obtained from the National Resident Matching Program.^2^

Historically, IC attracted some of the most ambitious and procedurally oriented trainees in medicine.^2^^,^^3^ The opportunity to save lives in the midst of acute myocardial infarction, perform high-impact procedures, and master advanced catheter-based therapies represented the pinnacle of cardiovascular practice. However, the field’s reputation for long hours, frequent call schedules, and limited flexibility has increasingly deterred new graduates. The 2025 SCAI statement acknowledged that “a record number of unfilled positions should prompt national dialog about how to make IC training and practice more sustainable.”^6^ The problem, therefore, is not a sudden disinterest in cardiovascular procedures but a systemic shift in what young physicians value and are willing to sacrifice.

## Causes of the problem

One of the foremost drivers of this declining interest is the imbalance between workload and incentives. Interventional cardiologists often do mean clinical work of 63 ± 14 hours per week, not including unpredictable overnight emergency calls for ST-elevation myocardial infarction (STEMI) cases and postprocedural complications.^4^^,^^7^ Although this intensity has always been inherent to the specialty, the compensation structure has not evolved to match these demands.^7^ Surveys from the American College of Cardiology (ACC) have shown that interventional cardiologists’ income has plateaued relative to other subspecialties that require similar training lengths but offer more controlled lifestyles.^8^, ^9^, ^10^ Meanwhile, EP, which also involves intricate procedures and advanced technology, has managed to maintain comparable compensation with significantly fewer overnight emergencies and better work–life integration.^5^ This contrast has made EP increasingly attractive to younger cardiologists seeking procedural complexity without sacrificing personal stability.^2^, ^3^, ^4^, ^5^

A second and deeply intertwined factor is burnout and lifestyle strain. The emergency-driven nature of IC imposes significant emotional and physical stress, with high-risk decision-making under time pressure. A survey published in *JACC: Cardiovascular Interventions* in 2023 reported that >50% of interventional cardiologists experience burnout symptoms, a rate higher than most other cardiology subspecialties.^4^ The unrelenting STEMI calls, the anxiety of potential complications, and the constant readiness required contribute to chronic fatigue and emotional exhaustion. Furthermore, interventional cardiologists frequently face substantial after-hour demands that extend beyond their primary procedural responsibilities. In many hospitals, IC physicians are often called in to perform tasks such as temporary pacemaker placement, a procedure that could be managed by electrophysiologists.^11^^,^^12^ Additionally, patients with any potential need for nonemergency interventions frequently generate IC consults rather than seeing the general cardiologist on call, who might be the more appropriate first responder.^12^ These patterns contribute to the perception of a more stressful call schedule and broader workload for IC fellows and practicing physicians, highlighting systemic factors that may influence fellowship attractiveness and burnout risk. Younger generations of physicians are increasingly unwilling to accept these conditions as the price of lifestyle–work balance.^4^^,^^7^ Instead, they prioritize flexibility, protected time, and family—friendly schedules—values that modern electrophysiology and imaging fellowships have better accommodated.

The economic burden and extended training pipeline further compound the issue. After completing medical school, residency, and a 3-year general cardiology fellowship, physicians pursuing IC must invest another year (or more, if pursuing structural training) in high-intensity procedural education. By the time fellows finish, many are in their mid-30s with significant educational debt, often exceeding $300,000.^13^ The perceived return on this investment has declined, particularly as hospital-based procedural reimbursements face ongoing reductions. Unlike prior decades, when the cath lab was the center of cardiovascular innovation and reimbursement strength, current health-care models increasingly emphasize preventive care, structural imaging, and noninvasive management. Consequently, the once-coveted “cath lab culture” has lost some of its appeal, especially in community-based practices where procedural volumes are declining.

Moreover, changes in case mix and procedural dynamics have subtly reshaped the field’s identity. Advances in pharmacology, early reperfusion systems, and preventive cardiology have led to a modest decline in STEMI volumes in many regions. Meanwhile, the field has diversified into subdomains such as chronic total occlusion, structural heart interventions, and peripheral vascular work—areas requiring additional specialized training. For many fellows, the growing subspecialization within IC makes the pathway feel longer and less predictable.

Another critical dimension is the lack of diversity and inclusivity in IC. Initiatives such as SCAI Women in Innovations (WIN) and the ACC Diversity and Inclusion Task Force are commendable, and their impact is appreciated; however, a significant gap persists.^14^ Despite a significant increase in women trainees in IC fellowships—from 6.3% in 2008 to 20.1% in 2022—the specialty continues to be culturally perceived as dominated by men, which is more likely multifactorial, including radiation exposure concerns, call intensity, and workplace culture, rather than purely physical demands.^15^^,^^16^ By comparison, in the same year, women comprised 15% of EP fellows.^16^

Another plausible and possible contributor to declining interest in IC fellowships is the evolving procedure and the fluctuating job market landscape. Early-career interventional cardiologists perform a low median annual PCI volume of 59 cases, comparable to non–early-career operators for 57 cases.^17^ However, they are caring for higher-acuity presentations, including STEMI and urgent PCI, with a higher predicted risk of mortality and bleeding compared with more experienced colleagues, with modestly worse outcomes.^17^ At these volumes, interventional cardiologists spend only about 20% of their professional time in the cath lab, with the remaining 80% devoted to general cardiology responsibilities, inpatient consultations, and imaging.^18^ This practice pattern may contribute to professional dissatisfaction among interventionalists who prefer to work in the cath lab after procedure-based training, which poses a future possible challenge for the hospitals in maintaining 24/7 STEMI response teams, leading to potential delays in time-critical interventions. Additionally, the academic ecosystem suffers when fellowship programs cannot sustain the trainee volumes needed for procedural innovation and clinical research. This imbalance may ultimately weaken the pipeline of future educators and innovators in the field.

Furthermore, the concomitant rise in temporary mechanical circulatory support and extracorporeal membrane oxygenation in patients whose chances of survival with meaningful recovery are marginal introduces an ethical challenge. Interventional cardiologists may feel pressured to perform PCI on patients with minimal survival prospects, which can increase the emotional toll on the operator.^19^

## Occupational hazards and radiation exposure

An often-underrecognized factor contributing to the declining interest in IC is the high level of occupational radiation exposure faced by operators.^20^ Interventional cardiologists experience among the highest cumulative radiation doses of any medical specialty, leading to documented risks such as cataract formation, brain and thyroid tumors, skin injury, and potential genetic damage.^21^ Although occupational dose limits are regulated and generally not exceeded, even chronic low-dose exposure carries biological risks that may accumulate silently over time, given the absence of a true threshold below which genetic effects do not occur.^22^

Although electrophysiologists once shared comparable exposure risks, the widespread adoption of three-dimensional electroanatomic mapping and multimodal imaging has significantly reduced reliance on fluoroscopy, allowing EP operators to perform many procedures with minimal or near-zero radiation exposure.^23^ This contrast has further contributed to EP’s growing appeal among cardiology fellows, who increasingly seek technologically advanced yet safer procedural environments.

Traditional radiation-protection measures—such as lead aprons, thyroid collars, and leaded goggles—have helped mitigate exposure but come with their own health burdens.^24^ Extended use of heavy lead aprons has been linked to orthopedic complications, including chronic back pain and spinal disorders.^24^^,^^25^ The combination of radiation risk and musculoskeletal strain has become a growing deterrent for younger physicians weighing the physical sustainability of a long interventional career.

## Proposed solutions

Addressing these challenges requires systemic reengineering in both training and practice environments. First, health-care systems and training institutions must reassess compensation models and call structures to reflect the realities of interventional practice. Implementing differential pay for high-acuity call coverage, expanding call-sharing networks, and providing protected rest periods could reduce burnout and enhance sustainability. Programs should also consider flexible fellowship models, allowing tailored training in coronary, structural, or peripheral interventions depending on career goals, thereby reducing redundancy and training fatigue. For example, adopting the supplemental compensation for STEMI calls suggested by Duffy et al^26^ could create a “win-win-win” situation—benefiting patients, hospitals, and physicians. Furthermore, incorporating downtime postcall would reduce the fatigue burden.

Second, fostering work–life integration should become a formal curricular goal. Wellness initiatives, structured mentorship, and mental health support systems must be embedded in fellowship programs rather than offered as optional add-ons. Interventional cardiology must evolve from a culture of endurance to one of resilience and balance. Mentorship programs, particularly those connecting fellows with early-career interventionalists who have successfully navigated similar challenges, can provide realistic guidance and emotional support. Additionally, incorporating simulation-based training could reduce procedural anxiety and enhance technical proficiency before high-stake clinical exposure.

Third, federal and institutional policy incentives can play a pivotal role in reshaping workforce distribution and motivation. Loan repayment or forgiveness programs for interventional cardiologists who commit to underserved regions would make the specialty more financially viable for trainees burdened by debt. Hospitals can also implement retention bonuses or long-term incentive plans for interventional cardiologists who assume heavy call responsibilities. Furthermore, professional societies like SCAI and the ACC should continue advocating for reimbursement reform that recognizes the time-critical, high-risk nature of IC procedures. These economic and structural reforms would not only attract applicants but also ensure equitable access to advanced cardiovascular care nationwide.

Fourth, perhaps one of the most transformative solutions lies in reimagining the cardiology training structure itself. The current 7-to-8-year postmedical school pathway–including 3 years of general cardiology followed by 1 or more subspecialty years—is increasingly viewed as inefficient. To address this, educators and professional societies should consider shortening general cardiology fellowship or residency training, allowing earlier entry into IC. Integrating procedural exposure and track-based training earlier during general cardiology can streamline skill acquisition while maintaining competency standards. Similar models in Europe and Canada demonstrate that earlier procedural engagement enhances technical proficiency without compromising patient outcomes. This change could reduce training fatigue, lessen educational debt, and make IC more competitive relative to other fields. By linking competency-based advancement with earlier hands-on experience, the field could attract younger, motivated candidates who seek both mastery and manageable career timelines.

In conclusion, the growing number of unfilled IC fellowships reflects a critical inflection point for the specialty. The same forces that once made the cath lab the pinnacle of medical heroism—intensity, immediacy, and technical mastery—now represent deterrents for a generation that values longevity, flexibility, and holistic well-being. Meanwhile, electrophysiology’s surge in popularity highlights that procedural medicine can thrive when lifestyle, compensation, and innovation are balanced thoughtfully. The future of IC will depend on its ability to adapt—to reshape its identity not through nostalgia, but through purpose, equity, and sustainability. The 2025 Match results are more than statistics; they are a call to action for the profession to realign its values with the evolving realities of modern medicine.

## Declaration of competing interest

The authors declared no potential conflicts of interest with respect to the research, authorship, and/or publication of this article.
